# Cryo-electron Microscopy Structure of the Acinetobacter baumannii 70S Ribosome and Implications for New Antibiotic Development

**DOI:** 10.1128/mBio.03117-19

**Published:** 2020-01-21

**Authors:** Christopher E. Morgan, Wei Huang, Susan D. Rudin, Derek J. Taylor, James E. Kirby, Robert A. Bonomo, Edward W. Yu

**Affiliations:** a Department of Pharmacology, Case Western Reserve University School of Medicine, Cleveland, Ohio, USA; b Department of Pathology, Beth Israel Deaconess Medical Center, Boston, Massachusetts, USA; c Harvard Medical School, Boston, Massachusetts, USA; d Department of Medicine, Case Western Reserve University School of Medicine, Cleveland, Ohio, USA; e Louis Stokes Cleveland Veterans Affairs Medical Center, Cleveland, Ohio, USA; f Department of Pharmacology, Case Western Reserve University School of Medicine, Cleveland, Ohio, USA; g Department of Molecular Biology and Microbiology, Case Western Reserve University School of Medicine, Cleveland, Ohio, USA; h Department of Biochemistry, Case Western Reserve University School of Medicine, Cleveland, Ohio, USA; i Case Center for Proteomics and Bioinformatics, Case Western Reserve University School of Medicine, Cleveland, Ohio, USA; j CWRU-Cleveland VAMC Center for Antimicrobial Resistance and Epidemiology, Cleveland, Ohio, USA; MedImmune

**Keywords:** 70S ribosome, *Acinetobacter baumannii*, cryo-EM, antibiotic resistance, structural biology

## Abstract

Antimicrobial resistance is a major health threat as it limits treatment options for infection. At the forefront of this serious issue is Acinetobacter baumannii, a Gram-negative opportunistic pathogen that exhibits the remarkable ability to resist antibiotics through multiple mechanisms. As bacterial ribosomes represent a target for multiple distinct classes of existing antimicrobial agents, we here use single-particle cryo-electron microscopy (cryo-EM) to elucidate five different structural states of the A. baumannii ribosome, including the 70S, 50S, and 30S forms. We also determined interparticle motions of the 70S ribosome in different tRNA bound states using three-dimensional (3D) variability analysis. Together, our structural data further our understanding of the ribosome from A. baumannii and other Gram-negative pathogens and will enable structure-based drug discovery to combat antibiotic-resistant bacterial infections.

## Abstract (precis)

Acinetobacter baumannii is a severe nosocomial threat largely due to its intrinsic antibiotic resistance and remarkable ability to acquire new resistance determinants. The bacterial ribosome serves as a major target for modern antibiotics and the design of new therapeutics. Here, we present cryo-EM structures of the A. baumannii 70S ribosome, revealing several unique species-specific structural features that may facilitate future drug development to combat this recalcitrant bacterial pathogen.

## INTRODUCTION

The ribosome represents a major target for antimicrobial agents as it is vital for protein synthesis and is one of the largest molecular machines in the cell (1). Although ribosomes are highly conserved among different living organisms, bacterial and eukaryotic ribosomes are nevertheless structurally distinct. For this reason, many antimicrobial agents, including macrolides, ketolides, lincosamides, oxazolidinones, aminoglycosides, and tetracyclines, selectively inhibit the function of bacterial ribosomes (2). As the ribosome is a large, complex molecular machine consisting of several large ribosomal RNAs and over 50 proteins, it offers many sites that are potentially druggable (3). However, to date, existing antimicrobial agents engage only a small fraction of this chemical space. Therefore, exploration of ribosome structure and function in a species of great clinical concern will allow us to define new targets for ribosome engagement and in turn enable discovery of novel therapeutic strategies to combat multidrug-resistant pathogens like Acinetobacter baumannii. Importantly, differences in ribosome structure among prokaryotes can potentially be exploited to develop therapies that selectively target pathogens and leave protective normal flora intact, thus avoiding subsequent therapy-associated colonization with resistant pathogens and morbid diseases such as Clostridium difficile colitis.

Bacterial 70S ribosomes consist of two subunits, which are the 50S and 30S (1, 4, 5). The large 50S subunit, which includes the 23S and 5S rRNAs and binds aminoacyl tRNA (aa-tRNA), catalyzes peptidyl transfer and participates in polypeptide elongation. The small 30S subunit, which includes 16S rRNA, recognizes mRNA, ensures decoding fidelity, and initiates protein synthesis. The mature 70S ribosome contains the so-called A, P, and E tRNA binding sites. The A site allows charged aminoacyl-tRNA that matches the mRNA codon to enter the ribosome. The P site docks peptidyl-tRNA that represents the elongating polypeptide chain. The E site engages the deacylated-tRNA, which exits the ribosome from this location. This complex machinery is currently targeted by several different classes of antimicrobial protein synthesis inhibitors. For example, macrolides and oxazolidinones engage the P site of the large 50S subunit and block peptidyl transferase and initiation, respectively, whereas many aminoglycosides and tetracyclines interact with the 30S subunits (3, 6).

To look into the possibility of identifying new antibiotic binding sites in bacterial ribosomes, we chose to elucidate the structure of the Acinetobacter baumannii 70S ribosome. A. baumannii has emerged as one of the most problematic and highly antibiotic-resistant superbugs (7). These bacteria exhibit a high level of multidrug resistance (MDR) to a broad range of antimicrobial agents. Of great concern, carbapenem resistance is often commonly found in clinical isolates, greatly limiting the availability of safe, effective options (8). There is also an increasing trend of resistance to last-resort antibiotics, such as colistin (9–12). Infections caused by these strains are untreatable. Complicating this already dire situation, A. baumannii strains are able to persist for long periods of time in patients and clinical settings, thus heightening the capacity of nosocomial spread (13). Currently, there is no empirical standard treatment regimen for Acinetobacter infections. Decisions on therapy are made on a case-by-case basis by a health provider based on available susceptibility data, where active options remain.

Here, we report cryo-electron microscopy (cryo-EM) structures of three different conformational states of the A. baumannii 70S ribosome at resolutions between 2.82 and 3.04 Å. We also present structures of the 50S and 30S subunits alone at resolutions of 2.95 Å and 4.40 Å, respectively. Our study reveals several unique structural features for this 70S ribosome. Therefore, our study identifies plausible targets for future drug development against A. baumannii infection.

## RESULTS AND DISCUSSION

### Structure of the A. baumannii 70S ribosome.

**FIG 1:**
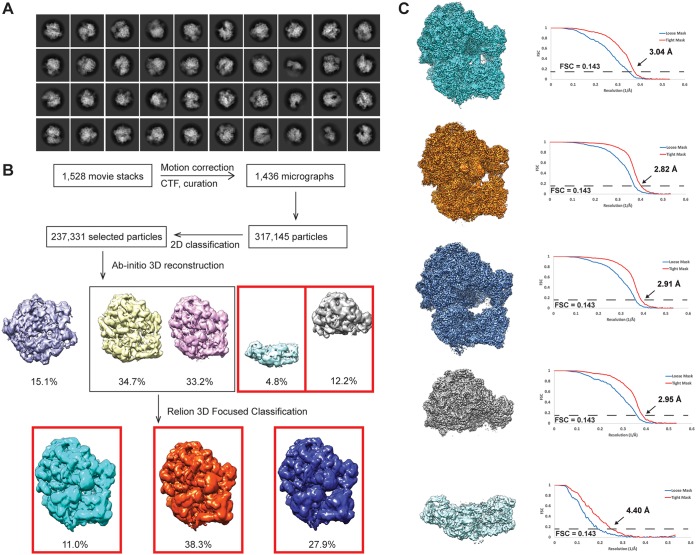
Cryo-EM processing of the A. baumannii ribosome data set. (A) 2D classification of the data results in high-resolution classes showing different views of the A. baumannii ribosome. (B) Extensive classification gives rise to five different ribosomal classes from this single data set. Maps in red boxes were selected for refinement. (C) High-resolution refinement of selected classes in cryoSPARC, version 2. Final classes consisted of (from top to bottom) P site occupied, E site occupied, empty, the 50S subunit, and the 30S subunit. Gold standard Fourier shell correlation (GS-FSC) curves depicting the resolution are included.

**FIG 2:**
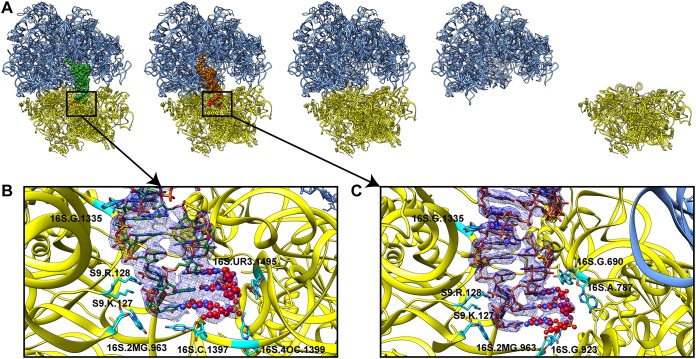
Cryo-EM structures of the A. baumannii ribosome. (A) Structures of the A. baumannii ribosome determined in this study: P site occupied, E site occupied, empty, 50S, and 30S. (B and C) In both the P-site-occupied and E-site-occupied structures, evidence for tRNA (green, P-site tRNA; brown, E-site tRNA) bound to mRNA (red; ball and stick representation) in the 30S subunit is present and modeled. Nearby residues are highlighted in cyan.

We used cryo-electron microscopy (cryo-EM) to determine high-resolution structures of the A. baumannii 70S ribosome. Extensive classification gave rise to five different classes within this single data set (Fig. 1). The first three classes depict structures of the mature 70S ribosome in the absence and presence of a tRNA molecule occupying the P or E site (Fig. 2). These three structures were designated P-site-occupied, E-site-occupied, and empty 70S ribosome, which were refined to resolutions of 3.04, 2.82, and 2.91 Å, respectively (Tables 1 and 2and Fig. 1). Like other bacterial ribosomes, the three A. baumannii 70S ribosome structures consist of both the 50S and 30S ribosomal subunits. The large 50S subunit contains 23S rRNA and 5S rRNA. In addition, because of the high-quality cryo-EM maps, we unambiguously observed 28 ribosomal proteins (r-proteins) in each 50S subunit of these 70S ribosomes. We also resolved the small 30S subunit of these three 70S ribosome structures. Based on the high-resolution cryo-EM maps, we identified the 16S rRNA and 20 r-proteins in each 30S subunit.

**TABLE 1:**
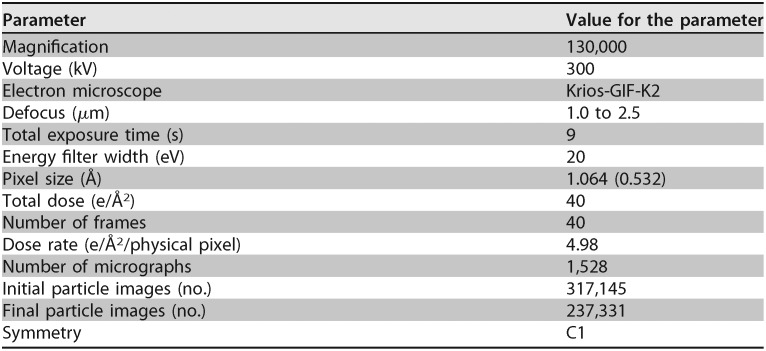
Cryo-EM data collection

**TABLE 2:** Cryo-EM model statistics

Model statistic^*a*^	Value for the structure				
	E site occupied	P site occupied	Empty	50S	30S
Refinement					
Resolution	2.82	3.04	2.91	2.95	4.4
FSC threshold	0.143	0.143	0.143	0.143	0.143
Map resolution range (Å)	2.39–30	2.32–30	2.35–30	2.62–30	2.26–30
Model cutoff (Å)	3	3.1	3	3	4.5
Model composition					
Nucleotides	4,615	4,615	4,535	3,007	1,528
Protein residues	5,457	5,458	5,457	3,117	2,311
RMSD					
Bond lengths (Å)	0.002	0.002	0.002	0.002	0.002
Bond angles (°)	0.484	0.460	0.479	0.448	0.434
Validation					
MolProbity score	1.48	1.45	1.49	1.48	1.65
Clash score	5.95	6.16	5.91	5.91	11.21
Poor rotamers (%)	0.07	0.00	0.05	0.04	0.05
Ramachandran plot (%)					
Favored	97.16	97.43	97.05	97.09	97.62
Allowed	2.76	2.52	2.89	2.81	2.38
Disallowed	0.07	0.06	0.06	0.1	0
CC_mask_	0.82	0.85	0.83	0.86	0.74
CC_box_	0.82	0.86	0.83	0.85	0.88
CC_vol_	0.81	0.83	0.82	0.85	0.74

a CC, correlation coefficient.

The fourth ribosomal class contains only the disassembled 50S subunit (Fig. 1 and 2). This structure was determined to 2.95 Å (Table 2). Again, we observed that this large 50S subunit was comprised of the 23S rRNA, 5S rRNA, and 28 r-proteins. The last ribosomal class depicts the disassembled 30S subunit structure. We determined this structure to a resolution of 4.40 Å (Table 2), where this small ribosomal subunit contains the 16S rRNA and 19 r-proteins (Fig. 1 and 2).

Within the P-site-occupied and E-site-occupied 70S ribosome structures, a tRNA was found to bind at the P site and E site, respectively. In addition, an extra density that resembles a small fragment of mRNA was observed in the vicinity (Fig. 2). As we did not add any tRNAs and mRNAs in our cryo-EM sample, these tRNA and mRNA fragments were probably copurified with the 70S ribosome. We therefore used a formylmethionyl (fMet)-tRNA and three-base mRNA fragment corresponding to the start codon for structural refinement. There is also evidence that the tRNA occupying the P site was aminoacylated compared with that of the previously solved E. coli 70S ribosome structure. Thus, an fMet molecule was included at the aminoacyl end of the P-site-bound tRNA (see Fig. S1 in the supplemental material).

10.1128/mBio.03117-19.1 FIG S1 Aminoacylated P-site tRNA. (A) P-site occupied structure of the A. baumannii 70S ribosome. (B) Expanded region showing density corresponding to an amino acid (modeled as fMet) at the aminoacyl end of the tRNA near the peptidyl transferase center (PTC) and exit tunnel. Download FIG S1, PDF file, 0.5 MB. Copyright © 2020 Morgan et al. 2020 Morgan et al. This content is distributed under the terms of the Creative Commons Attribution 4.0 International license.

### Conformational flexibility of the 70S ribosome.

**FIG 3:**
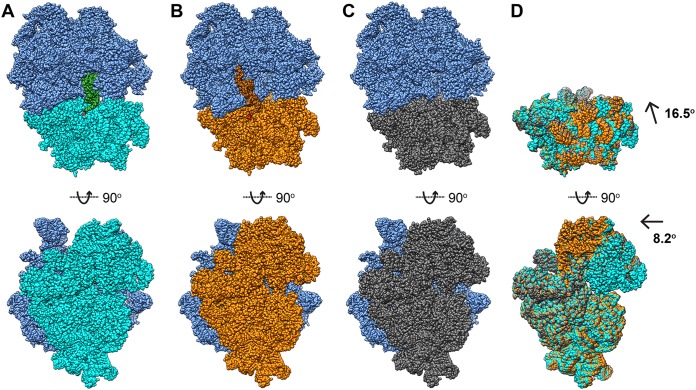
Dynamics of the A. baumannii ribosome. Comparison of the P-site-occupied, E-site-occupied, and empty structures depicts changes in 50S/30S orientation and 30S ratcheting state. (A) P-site-occupied 70S structure (blue, 50S subunit; cyan, P-site-occupied 30S subunit; green, P-site-bound tRNA). (B) E-site occupied 70S structure (blue, 50S subunit; orange, E-site occupied 30S subunit; brown, E-site bound tRNA). (C) Empty 70S structure (blue, 50S subunit; gray, empty 30S subunit). (D) 30S subunits shown after alignment of the 50S subunits for comparison. The 30S head is found to shift up to 16.5° while the 30S subunit is found to rotate about 8°.

In comparison with the P-site-occupied, E-site-occupied, and empty structures, there is only a subtle difference in the overall conformation of individual rRNAs and r-proteins. A pairwise superimposition of the 23S rRNAs from these three ribosome structures gives rise to a range of root mean square deviations (RMSD) between 2.0 and 2.6 Å. However, the relative orientations of the 50S and 30S subunits are quite distinct among these structures, along with the rotation of the 30S head between P-site-occupied, E-site-occupied, and empty structures (Fig. 3). As an active ribosome must go through a number of conformational states in order to synthesize proteins, we suspect that these three structures may simply reflect the conformation of various transient states of this 70S ribosome within the translation cycle although the process of translation is complicated and involves multiple tRNAs occupying different sites at a time (1, 14). Thus, the P-site-occupied structure may represent a transient state where the tRNA is bound within the P site and interacts with the mRNA trinucleotide. This state has been found to resemble a posttermination state (15). Subsequently, a deacylation and translocation process must occur. This process shifts the tRNA molecule accompanied with the mRNA to the E site and forms the E-site-occupied intermediate. Eventually, the bound tRNA and mRNA may exit the E site and form the empty conformation of the ribosome to complete the cycle. These two states likely represent important intermediates in ribosome recycling (15). Comparing the P-site-occupied, E-site-occupied, and empty structures, we observed that the small 30S subunit significantly alters its relative orientation in relation to the large 50S subunit of the ribosome (Fig. 3). This conformational change facilitates the movement of tRNA and mRNA to advance the translation cycle. Although our cryo-EM data do not include the A-site-occupied state of the 70S ribosome structure, these data do allow us to depict how 70S switches its conformation from the empty to P-site-occupied and E-site-occupied states and eventually go back to the empty state to complete the transition.

Upon examining the 70S structures, it is noticeable that there are significant differences in intersubunit interactions between the three conformations. Most notably, changes are seen in contacts between the A-site finger (H38 of the 23S rRNA) and r-protein S13 (30S subunit) alongside interactions between r-protein L5 (50S subunit) and r-protein S13 (16). These interactions are clearly seen in the E-site-occupied and empty structures with masked refinements. However, we cannot find these interactions in the P-site-occupied structure. This is likely due to the different orientations of the 30S subunit among the three different conformations (16) and is analyzed in detail with three-dimensional (3D) variability analysis (3DVA) below. In summary, the different 70S structures undergo global structural changes based on tRNA positioning, with the largest changes seen in motions of the 30S subunit and intersubunit connections.

### Species-specific features of A. baumannii 70S.

**FIG 4:**
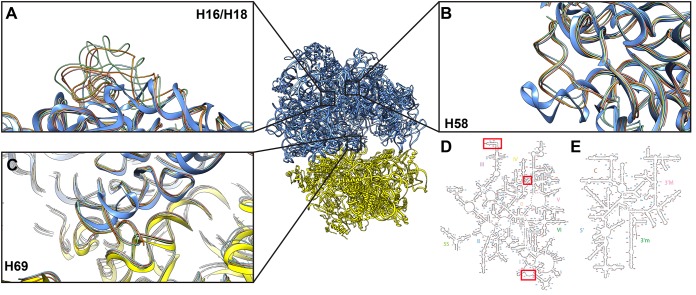
Structural features of the A. baumannii ribosome. (A) The H16/H18 region is highly variable in the model structures, with the A. baumannii ribosome (thick blue ribbon) having a significantly shorter region than the model structures (thin ribbon; cyan, E. coli; orange, S. aureus; green, M. tuberculosis; coral, B. subtilis). (B) H58 of A. baumannii is oriented differently than the model structures. (C) H69 of A. baumannii is in a different conformation than the model structures and does not form an intermolecular bridge with h44. (D) Secondary structure of the A. baumannii 5S and 23S rRNAs. Differences in structure (H16/H18, H58, and H69) marked with red boxes. (E) Secondary structure of A. baumannii 16S rRNA. Secondary structures created using the E. coli template (41).

In comparisons of cryo-EM structures from other bacterial 70S ribosomes, including those from Escherichia coli (17), Staphylococcus aureus (18), Mycobacterium tuberculosis (19), and Bacillus subtilis (20), we identified some important species-specific variations. First, there is a significant truncation within the H16/H18 region in the 23S rRNA of A. baumannii 70S (Fig. 4). This region is substantially longer in the E. coli and S. aureus ribosomes. In ribosome structures from M. tuberculosis and B. subtilis, additional helices are found in in this area, making this region even longer in these species. Given the fact that H16/H18 is highly variable and species specific, the unique structural information of A. baumannii 70S at this region could have a profound impact on future structure-guided drug discovery to combat this bacterial infection. Indeed, structural studies of the M. tuberculosis and Mycobacterium smegmatis 70S ribosomes suggested that there is a distinct difference in the conformation of H16 and neighboring r-protein L9 between these two ribosomes (19, 21), further emphasizing the importance of this region in relation to the specificity of different organisms.

Second, there is a significant conformational difference in helix H58 of the 23S rRNA of A. baumannii 70S compared with other ribosome structures (Fig. 4). Within the E. coli, S. aureus, M. tuberculosis, and B. subtilis structures, this helix universally makes a turn and positions itself to facilitate the interaction with H54/H55. Our cryo-EM map indicates that there is no such turn in the A. baumannii 70S structure, where H58 appears to form a straight helix and does not contact H54/H55. Interestingly, the helix H54 in M. tuberculosis 70S is quite specific, with a 100-nucleotide rRNA expansion (19, 21). The unique conformation of A. baumannii 70S within the vicinity of H58 and H54/H55 could be used as a good target for designing inhibitors to treat A. baumannii infection.

In addition, the conformation of H69 of the 23S rRNA, which is located in the vicinity of the P site, is quite distinct in the A. baumannii 70S structure (Fig. 4). H69 is presumed to interact with h44 of the 16S rRNA and form the intersubunit bridge B2a/d between the 30S and 50S subunits (16). This is evident from E. coli, S. aureus, M. tuberculosis, and B. subtilis 70S structures. However, our cryo-EM maps indicate that H69 in A. baumannii does not interact with h44, suggesting that this bridge may not, in fact, be essential for the assembly of the intact ribosome. Recently, a cryo-EM structure of the Pseudomonas aeruginosa 70S ribosome from an aminoglycoside-resistant clinical isolate was reported and suggested a similar possibility (22). In the structure of this resistant mutant, H69 presents a very unique conformation, which is bent toward 50S and also does not form an intersubunit bridge with h44 of the 30S subunit. This conformational state itself may interfere with the aminoglycoside binding site and in this way contribute to antibiotic resistance. The H69/h44 region is also an important target for the antituberculosis drug capreomycin (19). As the conformation of H69 of A. baumannii 70S is almost identical to that of aminoglycoside-resistant P. aeruginosa 70S, we suspect that the conformation of this helix in A. baumannii 70S may help the bacterium to resist aminoglycoside antibiotics. Our structures of A. baumannii 70S lay a solid foundation in the context of rational drug development.

### Docking of antibiotics to the 70S ribosome.

**FIG 5:**
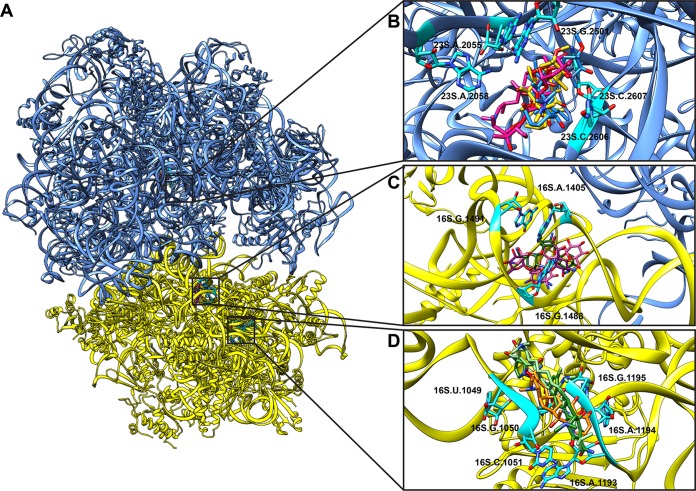
Antibiotic docking in the ribosome. (A) Three different antibiotic binding sites in the 70S ribosome. (B) The macrolide binding site (pink, azithromycin; yellow, clarithromycin; blue, erythromycin). (C) The aminoglycoside binding site (pink, amikacin; brown, gentamicin; purple, kanamycin). (D) The tetracycline binding site (orange, tetracycline; tan, tigecycline; green, eravacycline). Results for all classes represent known binding modes for the different antibiotics, showing that these binding sites are conserved in the A. baumannii ribosome.

The ribosome is known to bind a variety of antimicrobials. To elucidate how the A. baumannii 70S ribosome is capable of accommodating these agents, we used AutoDock Vina (23) to predict potential drug binding modes (Fig. 5). We chose three tetracyclines (tetracycline, tigecycline, and eravacycline), three macrolides (azithromycin, clarithromycin, and erythromycin), and three aminoglycosides (amikacin, gentamicin, and kanamycin) as they are known drugs that interact with bacterial ribosomes. We found that all three tetracyclines are bound within the location corresponding to the tetracycline binding site previously identified in the crystal structure of the Thermus thermophilus 30S subunit (24). The positions of the three bound tetracyclines partially overlap each other as the ribosome utilizes a similar set of residues to bind these drugs. Likewise, the three macrolide molecules are found to anchor at a location that corresponds to the macrolide binding site of the S. aureus 50S subunit (25), and the three aminoglycosides tested were found to bind in a region similar to the aminoglycoside binding site in the 30S subunit of E. coli (26). Again, we observed that the A. baumannii 70S ribosome employs a similar set of nucleotides to interact with all drugs. All antibiotics tested were found to bind favorably to their target sites (Table 3).

**TABLE 3:** Antibiotic docking studies

Drug class	Molecule	Binding affinity (kcal/mol)
Macrolide	Azithromycin	−7.4
	Clarithromycin	−7.1
	Erythromycin	−7.9
Tetracycline	Tetracycline	−6.9
	Tigecycline	−7.9
	Eravacycline	−8.0
Aminoglycoside	Amikacin	−7.8
	Gentamicin	−7.9
	Kanamycin	−7.3

### 3D variability analysis shows dynamic motions at different tRNA populations.

As we observed three distinct classes of various transient states of the 70S ribosome in a single data set, we decided to explore the detailed motions of these different populations using 3D variability analysis (3DVA) in cryoSPARC, version 2 (27). In each particle set, 3DVA revealed significant global motions between the large and small subunits. These motions are similar to those seen previously (28) and can be classified as rocking and rolling of the 30S subunit throughout each particle set.

The A-site finger (H38) of the 23S rRNA and r-protein L5 from the 50S subunit have been previously shown to form an important bridge with the 30S subunit (16). In the E-site-occupied and empty structures, interactions between the A-site finger and r-protein S13 were refined to high resolution with masked focused refinement. A884, U885, and C886 interact with a positively charged patch of residues on S13. This region is not present in the P-site-occupied tRNA particle pool, hinting that there may be significant dynamics in this region and that the interaction may be transient.

**FIG 6:**
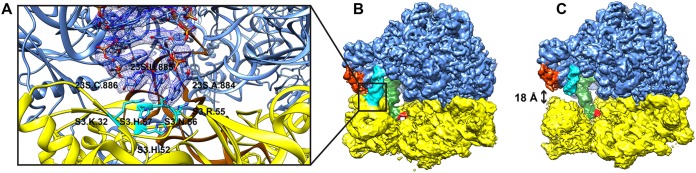
3D variability analysis of interparticle motions. (A) The interaction between H38 and r-protein S13 was resolved in the E-site structure but required masked refinement. (B and C) 3D variability analysis (3DVA) of the E-site particle set revealed that this is due to the transient nature of the interaction, where separation between H38 and S13 can reach up to 18 Å.

Studying the 3D variability analysis results of the E-site-occupied particle set showed that the interaction is indeed transient due to the extensive motions of the 30S head. The ratcheting and rolling motions of the 30S move the interaction sites of the A-finger helix and r-protein L5 with S13 up to 18 Å away (Fig. 6). This is in concert with a significant motion in the E-site-bound tRNA, which moves >7 Å throughout the particle set. These data show that that the low resolution of the A-site finger and L5 protein is due to the motions of the 30S head. Furthermore, the release of tRNA from the E site of the ribosome may require a concerted motion, with the 30S subunit moving away from the 50S subunit, allowing release of tRNA to the surrounding solution. Disrupting this dynamic interaction could be used for the development of new antibiotics.

Last, the ability of this technique to identify partially populated regions of the final EM map was examined (see Fig. S2 in the supplemental material). In the 3DVA movies from the E-site-occupied populated particle pool, a number of proteins that change in density quality over the course of the trajectory are seen. The most notable of these changes is r-protein S2, located in the 30S subunit between the core and head of the small subunit. To test if this was due to partial population, a focused mask was created for the protein, and focused 3D classification without alignment was performed in Relion, version 3. Indeed, results showed that only 60% of 70S particles contained this protein bound, while in the remaining 40% no S2 protein was present. A repeat refinement of the 30S with these particles resulted in significantly increased density quality, improving modeling results. The results show that this method can also be used to identify regions of significant variability, which can then be classified and refined.

10.1128/mBio.03117-19.2 FIG S2 3D variability analysis of partial populations. (A) Final map of the E-site populated ribosome. (B) Ribosomal protein S2 (cyan) refined to a notably lower resolution than the rest of the structure. This was revealed to be due to partial population through 3DVA. (C) Focused classification of this protein in Relion and reconstruction allowed for a complete density to be achieved. (D) The protein refined to a threshold and resolution similar to levels for the rest of the ribosome map. Download FIG S2, PDF file, 0.3 MB. Copyright © 2020 Morgan et al. 2020 Morgan et al. This content is distributed under the terms of the Creative Commons Attribution 4.0 International license.

In summary, our cryo-EM structures of the A. baumannii ribosome have allowed us to identify several unique static and dynamic subunit and substituent interactions. Taken together, these observations contributed to our understanding of shared and unique attributes of A. baumannii ribosomes that might be exploited for future development of ribosome-based therapeutics.

## MATERIALS AND METHODS

### Purification of ribosomes from A. Baumannii.

A. baumannii ribosomes were purified directly from A. baumannii strain AB0057. The cells were reconstituted in fresh ribosome lysis buffer (29) (20 mM Tris, pH 7.5, 50 mM magnesium acetate [MgOAc], 100 mM NH_4_Cl, 1 mM dithiothreitol [DTT], and 0.5 mM EDTA) and lysed using a French pressure cell. To pellet insoluble material, the crude lysate was centrifuged at 30,000 × *g* for 30 min. The resulting supernatant was then carefully layered on top of a sucrose cushion buffer (lysis buffer with 1.1 M sucrose) and spun for 16 h at 100,000 × *g*. This resulted in a ribosome-containing pellet that was stored at –80°C until further use.

A small portion of sample was reconstituted in ribosome resuspension buffer (20 mM HEPES, pH 7.6, 100 mM KCl, 5 mM MgOAc, 1 mM DTT, and 10 mM HN_4_Cl) and examined using negative stain to test for purity. While the sample was largely pure, there was a significant population of 50S and 30S ribosomal particles alongside the full 70S particles. Therefore, it was determined that a second purification step was favorable to remove some of the smaller subunits. This was accomplished through the use of sucrose gradient purification. Two sucrose buffers were prepared using ribosome buffer (20 mM HEPES, 14 mM MgOAc, 100 mM KCl, 0.2 mM DTT, and 0.1 mM phenylmethylsulfonyl fluoride [PMSF]) with 10% and 40% sucrose and mixed using a gradient maker. After the solution was cooled for >2 h, a portion of the crude ribosomal pellet was resuspended using ribosome buffer and layered onto the gradient and then centrifuged for 16 h at 100,000 × *g*. The resulting solution was fractionated by pushing a 60% sucrose gradient from the bottom of the tube, and fractions were analyzed using a plate reader. Fractions containing 70S ribosomal particles (largest peaks) were collected and exchanged into buffer containing 20 mM HEPES-KOH, 10 mM MgOAc, and 100 mM KCl using 100,000-molecular weight-cutoff Amicon centrifugal filters, flash frozen in liquid nitrogen, and stored at –80°C until further use.

### Electron microscopy sample preparation.

Sample quality was initially examined using negative stain cryo-EM using in-house microscopes (TF20 and T12) and standard protocols (30). Once sample quality was ensured, cryo-EM samples were prepared on Quantifoil R1.2/1.3 grids coated with graphene oxide (GO) prepared in-house. Ribosome samples (3.5 μl) at a concentration of 100 pM were frozen using an FEI Vitrobot with a 30-s wait time, followed by a blot of 10 s with a force of +5. Once the sample was frozen, sample quality was ensured using an in-house TF20 microscope and shipped for high-resolution data collection.

### Data collection and processing.

The NCI’s cryo-EM service was utilized for high-resolution data collection, providing 24 h of collection time in a Titan Krios (300 keV) equipped with a K2 Summit camera. Data were collected over 40 frames for 9 s at a nominal dose of 40 e/Å^2^ (4.98 e/s/physical pixel) with a GIF energy filter (20 eV). Data were collected in superresolution mode at defocus values between −1.0 and −2.5. Microscope nominal magnification was ×130,000, providing a physical pixel size of 1.064 Å/pixel (0.532 Å/pixel in superresolution). This provided 1,528 micrographs for data analysis.

All data were processed using a combination of cryoSPARC, version 2 (27), and Relion, version 3.0 (31). Movies were motion corrected using MotionCor2 (32), and Patch CTF was used for contrast transfer function (CTF) estimation (27). Particles were picked from 150 micrographs using the blob picker in cryoSPARC, version 2, binned by 4, and classified with two-dimensional (2D) classification to generate initial models for template picking. These templates were used to particle pick all micrographs, which were then binned by 4 and underwent two rounds of 2D classification (100 classes each) for particle cleaning. The selected high-quality classes resulted in ∼235,000 particles for further processing. This particle set was cleaned using *ab initio* reconstruction (5 classes), separating individual subunits (30S, 1 class; 50S, 1 class) from the assembled ribosomal particle (70S, 2 classes) and junk (1 class).

The three 70S classes were recombined and subjected to another round of *ab initio* classification using 8 classes to inspect the particles for any differences. Along with motions between the 50S and 30S ribosomal subunits as expected, three different tRNA populations were discovered in the single data set, P site occupied, E site occupied, and empty. To focus classification based on tRNA population, all particles were recombined and underwent *ab initio* reconstruction, followed by homogeneous refinement. At this stage, clear density for E-site tRNA was present while there was no clear density for P-site tRNA as the E-site tRNA population was found to be the most populated. Therefore, a mask of the E-site tRNA was created. The particle set then underwent 3D focused classification without particle alignment in Relion. Csparc2star.py from pyEM was used to create the .star file from .cs files. Separating populated and unpopulated particles, the unpopulated particles underwent another round of *ab initio* reconstruction followed by homogeneous refinement in cryoSPARC, showing clear density for P-site tRNA. The 3D focused classification process was repeated with this P-site tRNA, resulting in a populated and unpopulated class. This entire process resulted in the 3 classes solved, P-site-occupied, E-site-occupied, and empty ribosomal particles.

The particles were reextracted to full resolution and refined using homogeneous refinement in cryoSPARC. While the particle set was nominally high resolution, significant blurring of the 30S was seen due to interparticle dynamics between the 50S and 30S in the assembled particle, as expected (28). Therefore, focused local refinement was performed in cryoSPARC. The complete particle was split into three separated refinements based off motions: the 50S subunit, 30S core, and 30S head. Refinements were carried out with nonuniform refinement, a local shift search extent of 10 pixels, and a local rotation search extent of 15°. These showed significant improvement over consensus refinements and were therefore chosen to use for model building and refinement. As a final processing step, local resolution estimation and local filtering were performed in cryoSPARC.

### Model building and refinement.

Model fitting and refinement were performed in Coot (33) and Phenix (34). 23S, 16S, and 5S rRNAs were fit using the 2.9-Å cryo-EM structure of the E. coli ribosome (PDB accession number 5AFI) (17) as a starting model. While the ribosomes are fairly similar, there were some significant differences between the E. coli ribosome and the A. baumannii ribosome that made manual fitting difficult in some regions. Therefore, MDFF (35) from the NAMD (36) simulation suite were utilized to perform flexible fitting of the 23S and 16S rRNAs after manual mutation and fitting in Coot. Restraints were used to maintain secondary structure and torsional angles. Once the structure was complete, Coot was utilized to manually fit the rRNAs into the density. Modified nucleotides on the 23S and 16S rRNAs were then added based on the annotated enzymes from sequencing (37).

The 48 ribosomal proteins solved were homology modeled using the SWISS model server (38, 39), using other cryo-EM structures whenever possible. These were fit into the cryo-EM density using the E. coli ribosome structure (PDB accession number 5AFI) (17) as a model to guide placement. All proteins were manually fit into the density in Coot (33). In regions of lower resolution, the locally filtered density from cryoSPARC was used to guide placement and fit structures.

While not manually added during creation of the sample, tRNA was clearly present in the final densities and was therefore modeled. Since it is not possible to determine the exact tRNA that is present in the structure and also unlikely that there is only one tRNA since it came along from the cellular mixture, the A. baumannii Met-tRNA was modeled in its place. The P-site tRNA and E-site tRNA molecules from 5AFI were used as starting models and mutated to the A. baumannii fMet-tRNA sequence. Moreover, three residues from an mRNA were clearly present to pair with the anticodon of tRNA, and therefore this density was modeled as 5′-AUG-3′ to complement the Met-tRNA.

Mg^2+^ ions were manually added in Coot using the unmodeled blobs function at a threshold of 1.8 RMSD and the locally filtered maps to avoid placing atoms in noisy regions. After addition of Mg^2+^, water molecules were added using the “find waters” function in Coot. Two additional ions, Na^+^ and Zn^2+^, were also added.

Once modeled, structures were refined in Phenix (34). A single refinement was performed by combining the three focused refinements into a single map by using both the mask and vop add functions in Chimera. Phenix refinements were carried out using a weight of 0.7. After a single refinement, outliers were manually corrected in Coot (33), and the structure underwent a final refinement in Phenix, giving rise to the structures presented. Figures were created using Chimera (40).

### Antibiotic docking.

Docking experiments were performed with AutoDock Vina (23) using nine drugs from three different classes of antibiotics: macrolides (azithromycin, clarithromycin, and erythromycin), tetracyclines (tetracycline, tigecycline, and eravacycline), and aminoglycosides (amikacin, gentamicin, and kanamycin). Binding was focused to different regions on the 70S ribosome using a 20-Å box centered on previously determined binding sites (24–26). The ribosome was kept rigid while the antibiotics were flexible during the studies. Antibiotic poses were ranked according to free energy, and the most favorable mode and energy for each drug are reported in Fig. 5 and Table 3, respectively.

### 3D variability analysis.

To examine the overall flexibility and dynamics of the assembled ribosome, the three different structures, P-site occupied, E-site occupied, and empty, were studied using 3D variability analysis in cryoSPARC, version 2 (27). To save GPU memory, the 512-pixel full-resolution particles were down-sampled to a box size of 350 and underwent a single homogeneous refinement to create initial models. 3DVA was run by solving 3 modes over 20 iterations and filtering to 5 Å. The resulting 20 frames were visualized in Chimera (40).

### Data availability.

Atomic coordinates for the A. baumannii P-site-occupied, E-site-occupied, empty, 50S, and 30S ribosome structures have been deposited in the RCSB Protein Data Bank under accession codes 6V39, 6V3A, 6V3B, 6V3D and 6V3E and in EMDB under accession numbers EMD-21030, EMD-21031, EMD-21032, EMD-21033, and EMD-21034, respectively.
