# Supplementary material for: Cryo-electron Microscopy Structure of the Acinetobacter baumannii 70S Ribosome and Implications for New Antibiotic Development
Source: mBio. 2020 Jan 21;11(1):e03117-19. doi: 10.1128/mBio.03117-19 (PMC6974574; doi:10.1128/mBio.03117-19)
Supplement: FIG S1 [file mBio.03117-19-sf001.pdf]

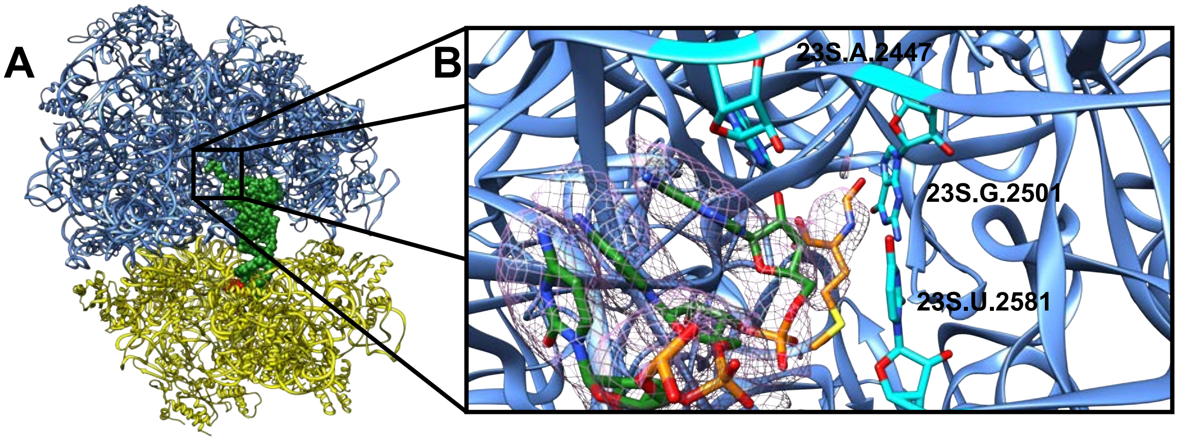

Fig. S1. Aminoacylated P-site tRNA. (A) “P-site occupied” structure of the *A. baumannii* 70S ribosome. (B) Expanded region showing density corresponding to an amino acid (modeled as fmet) at the aminoacyl end of the tRNA near the PTC and exit tunnel.
