# Supplementary material for: Cryo-electron Microscopy Structure of the Acinetobacter baumannii 70S Ribosome and Implications for New Antibiotic Development
Source: mBio. 2020 Jan 21;11(1):e03117-19. doi: 10.1128/mBio.03117-19 (PMC6974574; doi:10.1128/mBio.03117-19)
Supplement: FIG S2 [file mBio.03117-19-sf002.pdf]

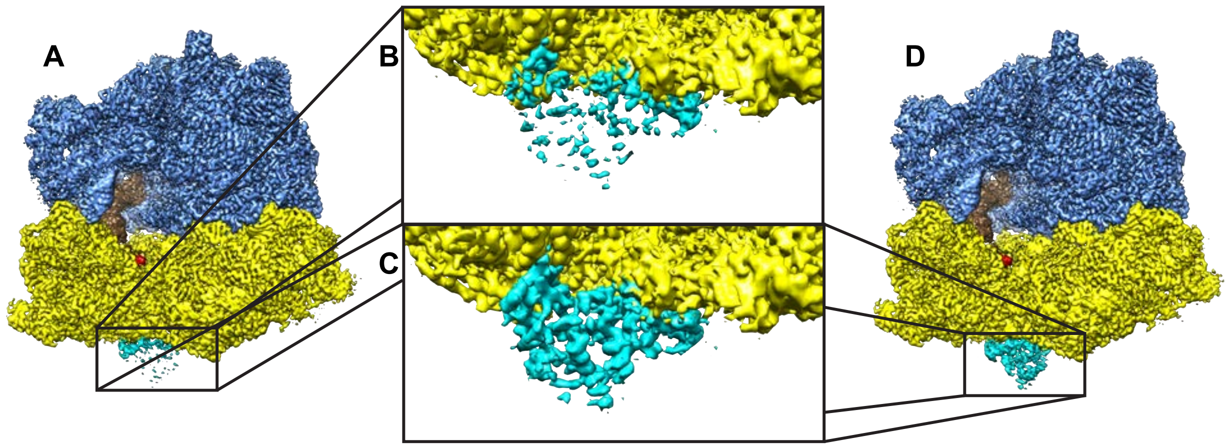

Fig. S2. 3D Variability Analysis of partial populations. (A) Final map of the "E-site populated" ribosome. (B) Ribosomal protein S2 (cyan) refined to a notably lower resolution than the rest of the structure. This was revealed to be due to partial population through 3DVA. (C) Focused classification of this protein in Relion and reconstruction allowed for a complete density to be achieved. (D) The protein refined to a similar threshold and resolution compared to the rest of the ribosome map.
